# Assembling an alkyl rotor to access abrupt and reversible crystalline deformation of a cobalt(II) complex

**DOI:** 10.1038/ncomms9810

**Published:** 2015-11-04

**Authors:** Sheng-Qun Su, Takashi Kamachi, Zi-Shuo Yao, You-Gui Huang, Yoshihito Shiota, Kazunari Yoshizawa, Nobuaki Azuma, Yuji Miyazaki, Motohiro Nakano, Goro Maruta, Sadamu Takeda, Soonchul Kang, Shinji Kanegawa, Osamu Sato

**Affiliations:** 1 Institute for Materials Chemistry and Engineering, Kyushu University, 744 Motooka, Nishi-ku, Fukuoka 819-0395, Japan; 2 Research Center for Structural Thermodynamics, Graduate School of Science, Osaka University, Toyonaka, Osaka 560-0043, Japan; 3 Department of Chemistry, Faculty of Science, Hokkaido University, Sapporo 060-0810, Japan

## Abstract

Harnessing molecular motion to reversibly control macroscopic properties, such as shape and size, is a fascinating and challenging subject in materials science. Here we design a crystalline cobalt(II) complex with an *n*-butyl group on its ligands, which exhibits a reversible crystal deformation at a structural phase transition temperature. In the low-temperature phase, the molecular motion of the *n*-butyl group freezes. On heating, the *n*-butyl group rotates ca. 100° around the C–C bond resulting in 6–7% expansion of the crystal size along the molecular packing direction. Importantly, crystal deformation is repeatedly observed without breaking the single-crystal state even though the shape change is considerable. Detailed structural analysis allows us to elucidate the underlying mechanism of this deformation. This work may mark a step towards converting the alkyl rotation to the macroscopic deformation in crystalline solids.

## Abstract (web summary)

Molecular motion can potentially be harnessed to control macroscopic properties such as size and shape. Here, the authors show that the phase transition of a cobalt(II) complex with an *n*-butyl group on its ligands, results in a reversible crystal deformation at the structural phase transition temperature.

## Graphical abstract

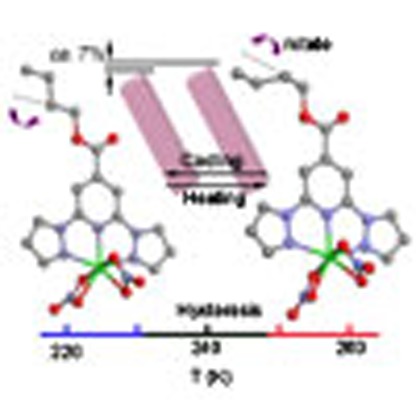

The development of new materials that exhibit reversible macroscopic changes in response to external stimuli has attracted significant attention for their potential applications in actuators and their mimicry of muscle cells^1^,^2^,^3^,^4^,^5^. To date, numerous molecular systems that undergo reversible molecular-level motions have been developed; however, it remains extremely challenging to amplify these motions to the macroscopic regime attained through synergy^6^,^7^,^8^,^9^,^10^. The lack of research in this area can possibly be attributed to the subtleties of molecular motion in solids, particularly in crystals. In fact, the crystal is still an ideal medium to study the relationship of molecular movements and macroscopic properties because of its close packing potentially, enabling macroscopic changes via molecular cooperation^11^,^12^,^13^.

As one of the typical molecular motions, rotational isomerization around the carbon–carbon bond has been widely studied^14^. Alkanes and alkyl derivatives, such as polyethylene, liquid crystals, biological membranes, and variously modified proteins are classical examples that undergo this type of isomerization, which is a major factor in the dynamics and activity of their molecular structures^15^,^16^,^17^. Recently, molecular crystals based on alkyl derivatives that exhibit conformational polymorphism have also been studied^18^,^19^. However, studies on converting alkyl rotation to reversible and large motion of the macroscopic crystal have never been reported.

With the above in mind, we turned our focus to alkyl derivatives—the potential rotors. Herein, we report a crystalline Co(II) complex containing an *n*-butyl derivative, which undergoes an entropy-driven structural phase transition and exhibits reversible macroscale deformation that changes with temperature. At the high-temperature phase the frozen conformation of the alkyl chain melts and undergoes the rotational isomerization accompanied by an increase in repulsion between adjacent molecules. This results in significant expansion along the long axis (*c* axis) of the crystal attained by synergy with π–π interactions.

## Results

### Preparation of crystalline complex 1

The cobalt(II) complex with a *n*-butyl group in its ligand, [Co(NO_3_)_2_(L)] (complex **1**), was synthesized by layering an acetone solution of a planar tridentate N-containing ligand (L=*n*-butyl-2,6-di(1*H*-pyrazol-1-yl)isonicotinate) on an acetone solution of Co(NO_3_)_2_·4H_2_O in a glass tube; the complex was successfully obtained as prismatic purple crystals at room temperature (see Methods).

### Characterization of the phase transition

**Figure 1:**
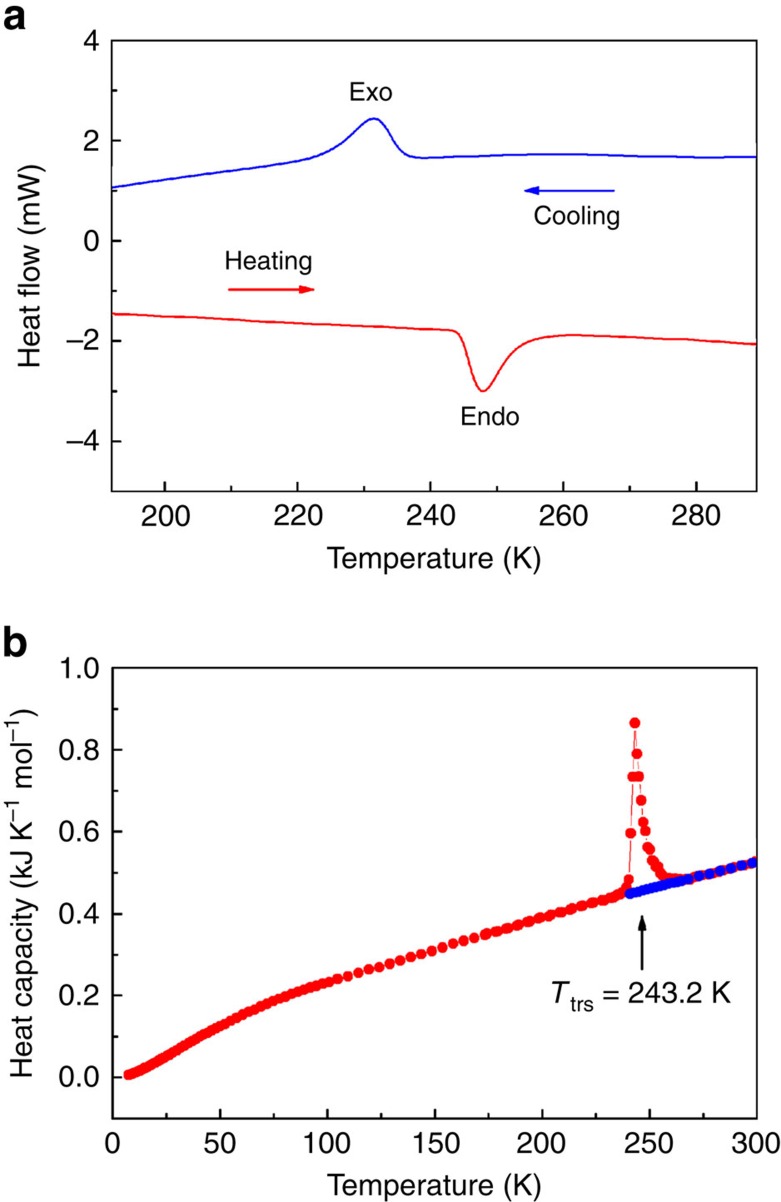
Temperature dependence of differential scanning calorimetry and heat capacity for 1. (**a**) DSC curves of crystal **1** recorded at the rate of 10 K min^−1^ during a cooling–heating (blue-red lines) cycle. Exo: exothermic peak; Endo: endothermic peak. (**b**) The phase transition temperature (*T*_trs_) is determined to be 243.2 K on heating. Filled red and blue circles represent the data obtained during heating mode after the samples were cooled to 7.6 and 240.9 K, respectively.

The occurrence of a thermal phase transition in **1** was confirmed by differential scanning calorimetry (DSC) measurements (Fig. 1a and Supplementary Fig. 1). The DSC curves for the polycrystalline **1** sample exhibit a single exothermic peak at 231 K during the cooling process; this peak corresponds to the transition to the low-temperature phase. In contrast, the DSC traces of **1** exhibit an endothermic peak at 248 K during the heating process. The relatively large thermal hysteresis of ca. 17 K and the distinct peak indicate a first-order phase transition.

### Structural characterization

**Figure 2:**
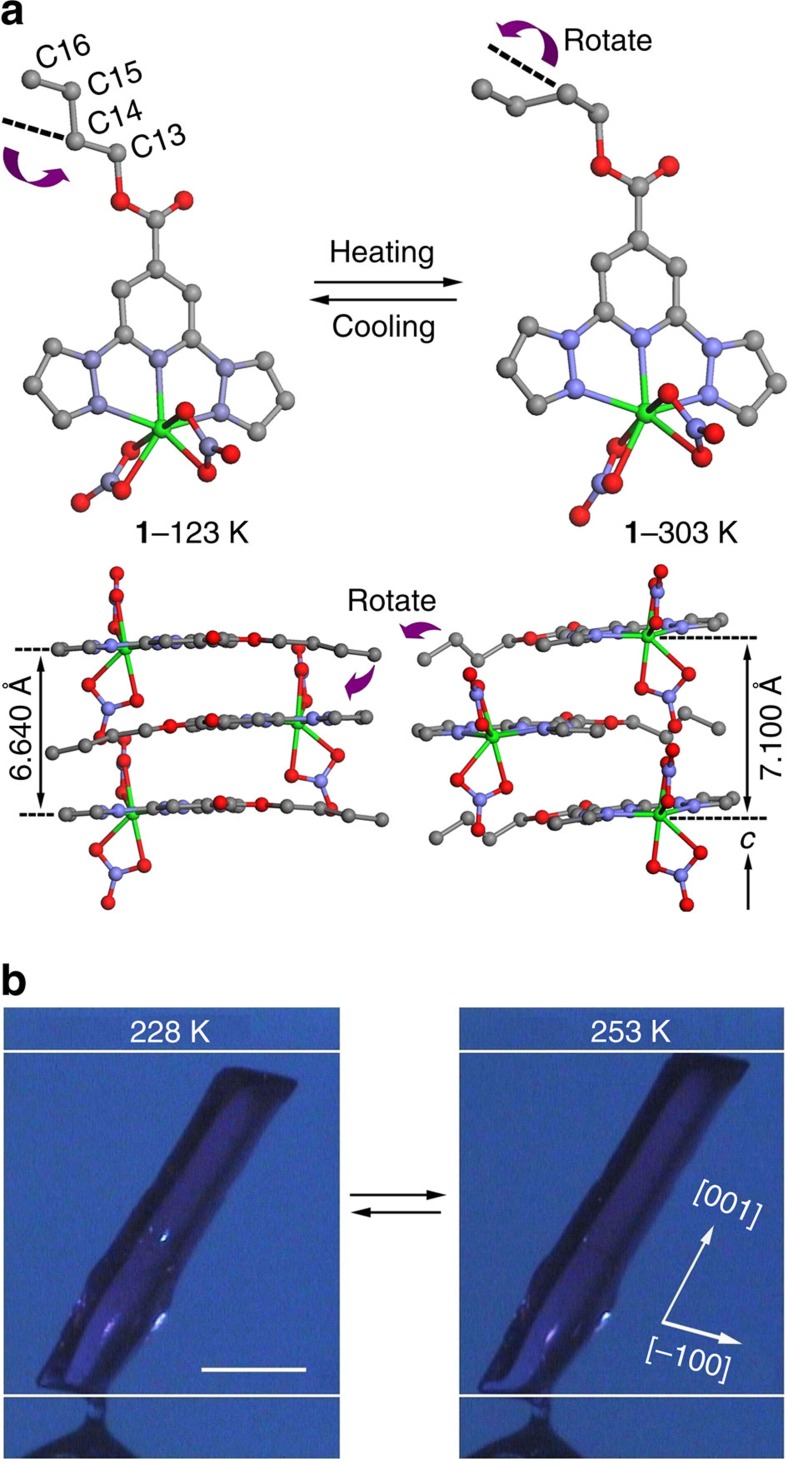
Molecular structures of crystal 1 in the low- and high-temperature phase. (**a**) The cobalt ions are coordinated by one tripodal L ligand and two nitrate anions in both phases. The molecules stack in parallel along the *c* axis with different distances between molecules in the low- and high-temperature phases. The *n*-butyl group of the ligand rotates ca. 100° around the C13–C14 bond following phase transition. Green, Co; grey, C; blue, N; red, O. Hydrogen and disordered carbon atoms (C14′ and C15′) are omitted for clarity. (**b**) The crystal length changes from 1.78 to 1.90 mm along [001] from 228 to 253 K and switches back to the original state upon cooling, scale bar represents 500 μm.

The crystal analyses of **1** were performed at 123 K (low-temperature phase) and at 303 K (high-temperature phase; Supplementary Table 1). The complex at 123 K crystallizes in a monoclinic space group *P*2_1_/*c*, with unit-cell parameters of *a*=9.794(3) Å, *b*=27.475(9) Å, *c*=7.548(3) Å and *β*=100.433(8)°; the asymmetric unit cell consists of one complex molecule. The cobalt ion is coordinated in a distorted pentagonal bipyramid geometry by three nitrogen atoms of the L ligand and four oxygen atoms of two NO_3_^−^ anions. The two NO_3_^−^ anions are located on both sides of the plane defined by the cobalt ion and the L ligand. The ligand L with a *n*-butyl group exhibits an *anti* conformation, without disorder. When the temperature was increased, a structural phase change occurred. The crystal structure at 303 K shows that **1** has the same space group as that at 123 K but different unit-cell parameters; the cell parameters at 303 K are *a*=9.8533(13) Å, *b*=25.995(3) Å, *c*=8.1782(10) Å and *β*=100.741(2)°. The ligand L now exhibits a *gauche* conformation and disorder, suggesting the occurrence of a *gauche*–*anti* transition through rotational isomerization^14^,^20^ (Fig. 2a and Supplementary Figs 2a and 3a). The *gauche* conformation, which is denoted by *gauche* **1**, is shown in Supplementary Fig. 3a. The variable-temperature infrared absorbance spectra of **1** support the induction of a structural phase transition (Supplementary Fig. 4) involving dynamic disorder of the *n*-butyl group^20^,^21^. The dynamic disorder of the *n*-butyl group in **1** is supported by the solid-state ^13^C CP/MAS NMR (magic-angle-spinning nuclear magnetic resonance) spectrum of [Zn(NO_3_)_2_(L)] (complex **2**; Supplementary Figs 5–7 and Supplementary Table 2) as an analogue to **1**, where the Co^2+^ is replaced with diamagnetic Zn^2+^, and by the solid-state ^2^H NMR spectrum of [Zn(NO_3_)_2_(L-*d*_9_)] (complex **2′**; Supplementary Figs 8–11 and Supplementary Table 3)^22^,^23^.

**Figure 3:**
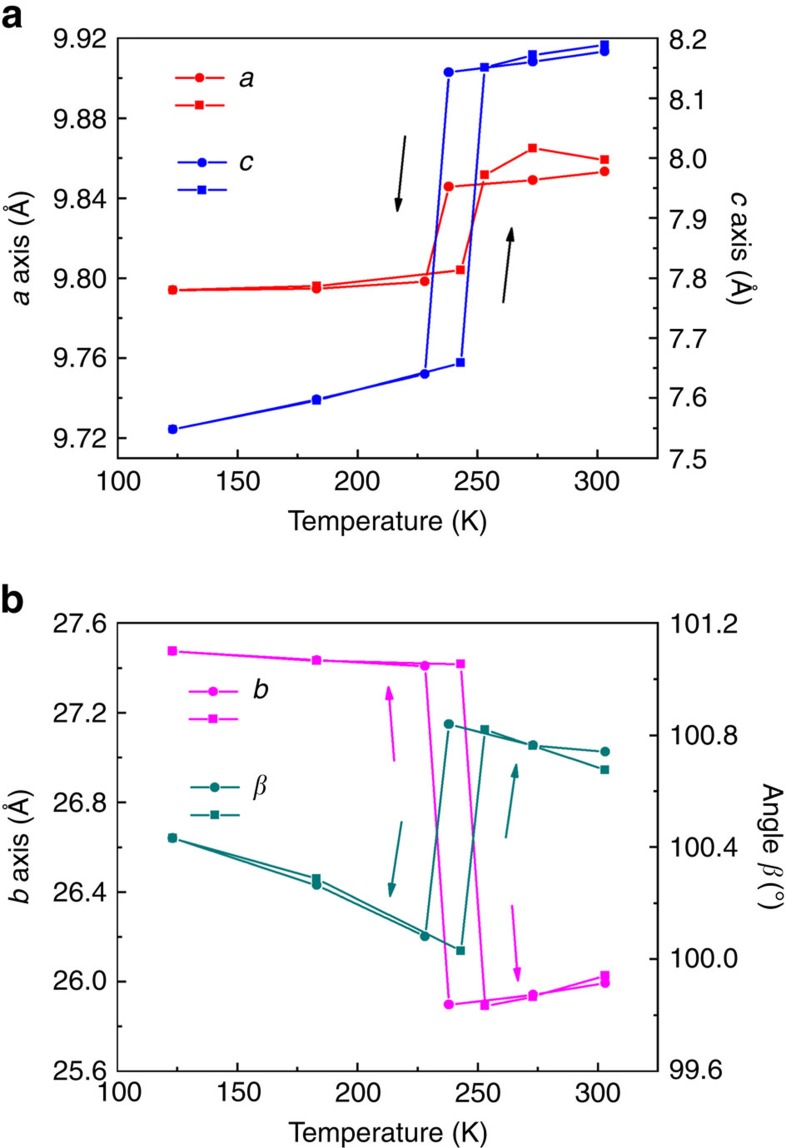
Cell metrics as a function of temperature. Variable-temperature single-crystal X-ray diffraction data of complex **1** were repeatedly collected for the same crystal in the range 123–303 K (heating, filled squares; cooling, filled circles). (**a**) Changes in the crystallographic *a* and *c* axes. (**b**) Variations of the angle *β* and the crystallographic *b* axis. The length of the *b* and *c* axes exhibit a reversible and abrupt change at ∼240 K and the percentage change is ∼5, respectively.

To investigate the structural change in detail, we measured the temperature-dependent variation in length of the three crystallographic axes and variation of the angle *β* (Fig. 3 and Supplementary Table 1). The unit-cell parameters undergo abrupt changes at temperatures below 238 K and above 243 K during cooling and heating, respectively; these changes correspond to the occurrence of phase transition, which is consistent with the DSC experimental results. As confirmed by the unit cells measured in the temperature range 123–303 K, we established that the transition between the two phases is reversible, with hysteresis. Whereas thermal expansion along the *a* axis and *c* axis is positive, a negative thermal expansion occurs along the *b* axis^24^.

### Thermal-induced deformation

**Figure 4:**
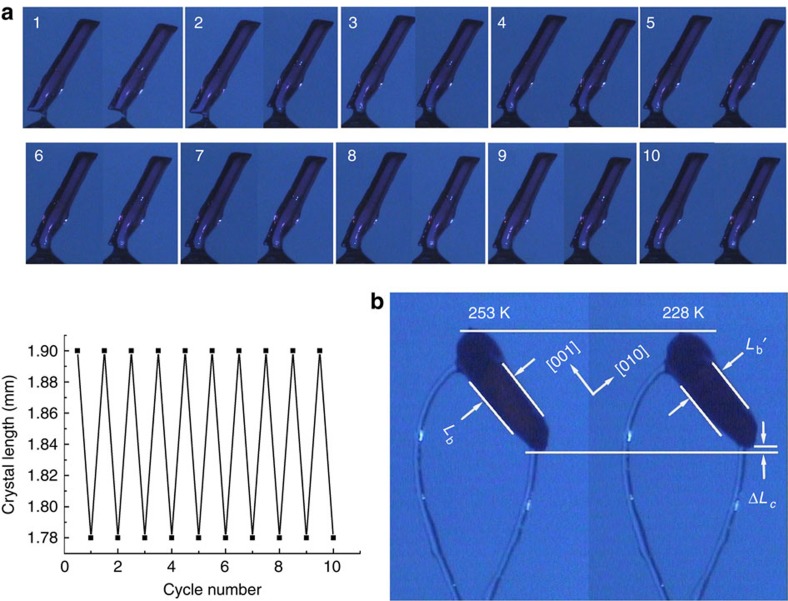
Reversible shrinkage and expansion of crystal 1. (**a**) Reversible shrinkage and expansion of the prismatic crystal on alternate heating and cooling at a rate of 5 K min^−1^ in the range 228–253 K. The crystal was glued onto a nylon loop and enveloped in a temperature-controlled stream of dry nitrogen gas. The crystal exhibits shrinkage and expansion along the long axis of the crystal in response to temperature. The reversible changes were recorded for 10 cycles without breaking the single-crystal state. (**b**) The increase in crystal size from ∼0.20 (*L*_*b*_) to 0.21 mm (*L*_*b*_*′*) along the [010] direction, and the contraction by ∼0.05 mm (Δ*L*_*c*_) (from about 0.83 to 0.78 mm) during cooling from 253 to 228 K.

Because of the structural phase transition, the prismatic crystal significantly contracts and expands reversibly during cooling and heating. The change is as large as ∼7% along the *c* axis and 5% along the *b* axis (Figs 2b and 4). The reversible crystal deformation was recorded as two videos (Supplementary Movies 1 and 2). As shown in the Supplementary Movie 1, the transformation was complete in <1 s when the cooling rate was 5 K min^−1^; the crystal appears to bend slightly like a snake during the contraction process. Moreover, as shown in Fig. 4, a crystal up to 1.9 mm in size exhibits no evidence of appreciable fatigue after 10 cycles, which is distinguishable from the behaviour of normal molecular crystals^11^,^12^,^25^,^26^,^27^. Reproducible crystal deformation is an essential property for practical applications and gaining insight into the structure–property relationship.

### Magnetic properties

Notably, the structural phase transition involves changes not only in crystal shape but also in magnetic properties. The magnetic susceptibility of microcrystal **1** was measured under heating and cooling modes in the temperature range 2–300 K; a step is observed at ∼245 K (Supplementary Fig. 12a). The change in magnetization is consistent with the phase transition results. The *χ*_m_*T* values below and above the transition temperature are 2.30 and 2.36 cm^3^ K mol^−1^, respectively. Magnetic anomaly can be explained as a result of the modulation of the quenching of the orbital angular momentum, which is related to the coordination environment of the cobalt ion^28^. For complex **1**, two significant changes occur in the environment of the cobalt ion before and after the phase transition: a nitrate ion is twisted along the dihedral angle N3–Co1–O6–N7 (α) from 143.43 to 150.40°, and the bond length Co1–O3 changes from 2.198 to 2.342 Å (Supplementary Fig. 12b and Supplementary Table 4).

## Discussion

**Figure 5:**
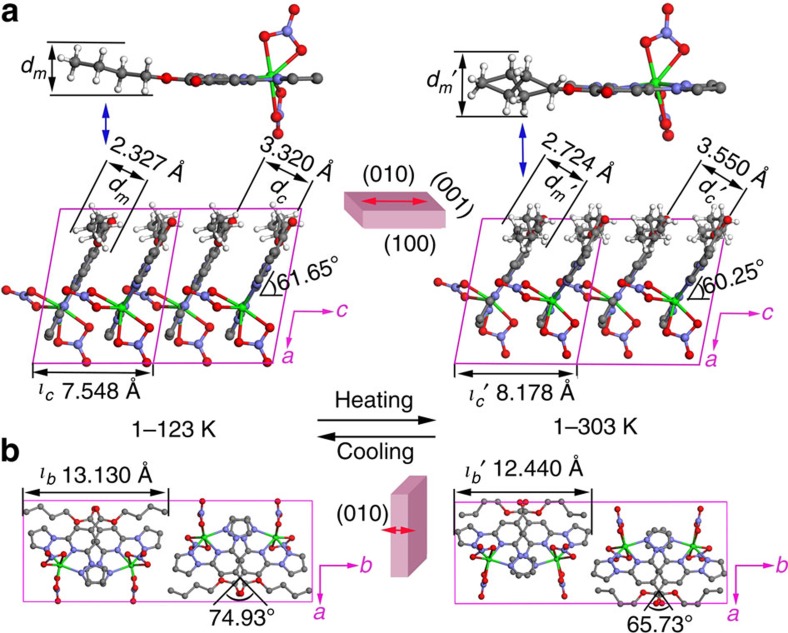
Molecular packing of a crystal of 1. Crystalline packing of molecules of **1** in the low- and high-temperature phase (**1**—123 K and **1**—303 K) as viewed from (**a**) the (010) face and (**b**) the (001) face. From the low-temperature phase to the high-temperature phase (**a**) the size of the *n*-butyl group in the direction perpendicular to the molecular plane (*d*_m_), the average distance between the molecules along the *c* axis (*d*_*c*_) and the length of the crystal *c* axis (*ι*_*c*_) increase to 2.724 Å (*d*_m_*′*), 3.550 Å (*d*_*c*_*′*) and 8.178 Å (*ι*_*c*_*′*), respectively; the tilt angle *ϕ* (Supplementary Fig. 2) decreases from 61.65 to 60.25° (*ϕ′*, Supplementary Fig. 3); (**b**) the size of each molecular column decreases from 13.130 to 12.440 Å (*ι*_*b*_→*ι*_*b*_*′*) along the *b* axis; and the crossing angle *ω* (Supplementary Fig. 2) decreases to 65.73° (*ω′*, Supplementary Fig. 3). The outlines of the crystal morphology and red arrows in the middle of the diagrams show the direction of contraction and expansion of the crystal, respectively. Green, Co; grey, C; white, H; blue, N; red, O.

We rationalize the origin of the extraordinarily large thermal deformation for crystal **1** at the molecular level by systematically comparing the crystal structures before and after the phase transition. In the low-temperature phase (123 K), the molecules stack in columns along the crystallographic *c* axis, as shown in Supplementary Fig. 2a; within each column, they are tilted such that their molecular planes (The plane is defined by the atoms N1, N3 and N5.) form an angle *ϕ* of 61.65° relative to the stacking direction [001], which is the long axis of the prismatic crystal. The adjacent molecules in each column are parallel, with an average distance *d*_*c*_ of 3.320 Å (Fig. 5a), and arrange in the scissor-crossover mode with a crossing angle *ω* of 74.93° (Supplementary Fig. 13). The column structure can be stabilized by π*–*π interactions and shape-complementary van der Waals interactions between interdigitated neighbouring π-conjugated molecules (Supplementary Fig. 14a). The three-dimensional molecular packing is primarily dictated by C–H···O nonconventional hydrogen bonds (Supplementary Fig. 2b), which play an important role in the phase transition and stabilization of the overall structure^28^; the hydrogen bond distance ranges from 3.201 to 3.281 Å.

In the high-temperature phase (303 K), the molecules stack in the same modes as in the low-temperature phase (Fig. 5a and Supplementary Fig. 3b), with a tilt angle *ϕ′* of 60.25° relative to the stacking direction and a crossing angle *ω′* of 65.73°; these angles are smaller than the corresponding angles (*ϕ* and *ω*) in the low-temperature phase. The average distance between the neighbouring molecules increases from *d*_*c*_=3.320 Å in the low-temperature phase to *d*_*c*_*′*=3.550 Å in the high-temperature phase on heating to reduce the repulsive interactions from the adjacent molecules along the *c* axis because the disordered *n*-butyl groups induced by rotational isomerization occupy more space. The repulsive interactions are mainly from the interactions of the *n*-butyl group with the molecular planes and coordinated nitrate ions of adjacent molecules (Supplementary Fig. 14b). Actually, the size of the *n*-butyl group in the direction perpendicular to the molecular plane increased from *d*_m_=2.324 to *d*_m_*′*=2.724 Å. The changes in the angle *ϕ* and in the distance between adjacent molecules result in an increase in the length of the crystal *c* axis from *ι*_*c*_=7.548 in the low-temperature phase to *ι*_*c*_*′*=8.178 Å in the high-temperature phase, and the crystal correspondingly extends ∼7% (≈{(*d*_*c*_*′* sin *ϕ*)*/*(*d*_*c*_*sin* *ϕ′*)−1} × 100%) along the long axis. In this transition process, the crossing angle *ω* decreases, which can be accounted for by a reduction of the repulsive interactions that arise from a decrease in the distance between the neighbouring nitrate ions and *n*-butyl groups after the rotational isomerization. As the rotational isomerization of *n*-butyl groups occurs, the size of each molecular column along the *b* axis decreases from *ι*_*b*_=13.130 to *ι*_*b*_*′*=12.440 Å, corresponding to the change in the crystal *b* axis from 27.475 to 25.995 Å (Fig. 5b), and the crystal contracts by ∼5% (≈{1–(*ι*_*b*_*′*/*ι*_*b*_)} × 100%), which is not easily observed because of the small crystal size in this direction. Because of the synergetic effect between the angles *ϕ* and *β*, the *a* axis crystal parameter does not significantly change between the two phases. After the phase transition, the hydrogen bonds change slightly and the π–π interactions are weakened by an increase in *d*_*c*_ to 3.550 Å (Supplementary Figs 3c and 14a).

To obtain information about the phase transition, we performed heat capacity measurements using an adiabatic calorimeter^29^ (Fig. 1b). The molar heat capacities under constant pressure (*C*_p_) showed a sharp peak accompanying a latent heat at 243.2 K, which is due to a phase transition, and also exhibited a supercooling phenomenon. These facts provide evidence of a first-order phase transition. Cooperative interaction plays an essential role in the phase transition. The transition enthalpy and entropy estimated from the heat capacity are Δ*H*=2.579±0.028 kJ mol^−1^ and Δ*S*=10.50±0.11 J K^−1^ mol^−1^, respectively. From Boltzmann's equation Δ*S*=*R* ln *N*, where *N* represents the ratio of possible conformations and *R* is the gas constant, the *N* value is ∼4 (*N*=3.54≈4). Careful investigation of the structure shows that the *n*-butyl group in **1** is completely ordered in the low-temperature phase. However, two methylene carbon atoms, that is, C14 and C15, are disordered in the high-temperature phase, in which each carbon atom has two possible sites (Δ*S*=*R* ln2^2^). These results suggest that the disordering of the two methylene carbon atoms is mainly responsible for the entropy gain in the phase transition. The *N* value of 4 suggests that the nature of the dynamic disorder motion is reorientational rather than free rotational. Furthermore, the entropy derived from crystal expansion should also contribute to the entropy gain^30^.

As previously discussed, the *n*-butyl group of the cobalt(II) complex is in the *anti* conformation in the low-temperature phase, and in the *gauche* conformation in the high-temperature phase. We performed periodic density functional theory calculations to investigate the shrinkage and expansion of the crystal. First, we optimized the low-temperature crystal structure; the lattice parameters as well as atomic coordinates were relaxed without symmetry constraints. The calculated unit-cell parameters are in good agreement with the experimental data (Supplementary Figs 15 and 16). We manually altered the structure of the *n*-butyl group from the *anti* to the *gauche* **1** conformation, and the system was optimized while the unit-cell parameters were kept fixed. In the optimized structure, the *n*-butyl group experiences a significant steric interaction with an adjacent Co(II) complex, which is likely to increase the distance between the neighbouring Co(II) complexes. The relative energy of 24.5 kJ mol^−1^ decreased to 7.5 kJ mol^−1^ when the unit-cell parameters measured from the *anti* conformation were relaxed. The steric interaction between the *n*-butyl group in the *gauche* conformation and the neighbouring Co(II) complexes is reduced in the course of the unit-cell-parameter optimization (Supplementary Fig. 17), which indicates that the repulsion is a driving force of the change of the crystal size. The obtained unit cells show an expansion of 7.7 % along the *c* axis and a shrinkage of 5.4 % along the *b* axis in the *anti*–*gauche* transition, which is also in good agreement with the experimental data.

Consequently, compound **1** exhibits a thermally induced structural phase transition at ∼240 K; the high-temperature phase has relatively larger volume per molecule, which results in a decrease of the stabilization energy derived from non-covalent interactions between constituent molecules (electro-static and π–π interactions) after the transition from the low-temperature to the high-temperature phase. The large increment of the enthalpy of the high-temperature phase relative to that of the low-temperature phase in the first-order phase transition is compensated by the gain in entropy that originates from the conformational change of the alkyl chain. These changes in orientation at the molecular level are amplified to the deformation at the micrometre scale in the crystal because of the collective motion of molecules in the whole crystal through cooperative interactions.

An important characteristic of **1** is that the thermally driven contraction and expansion of the crystal could be repeatedly observed. It has been reported that the organic compounds exhibiting crystal bending has anisotropic packing; there should be one strong interaction in a direction and a weaker interaction in a nearly perpendicular direction^31^. Careful investigation of the molecular interactions suggests that compound **1** has a one-dimensional nature in structure and that one set of interactions (π*–*π stacking) along the *c* axis is substantially stronger than those in a nearly perpendicular direction. The characteristic anisotropic molecular interaction is thought to effectively release the mechanical stress in **1** during the phase transition, resulting in the successful observation of a repeatable crystal deformation.

As described above, the Zn(II) analogue (complex **2**) exhibits the very similar structural change to **1** (Supplementary Figs 5 and 6 and Supplementary Table 2), though the phase transition temperature of **2** is different from that of **1**. On the other hand, when the pentyl, hexyl and decyl groups were used to replace the butyl group in **1**, the reversible transition was not observed in the Co(II) complexes (complexes **3**–**5**). The crystal structures of these complexes are different from that of **1**, including the conformation of the alkyl chain and molecular stacking in the crystals (Supplementary Fig. 18 and Supplementary Table 2). These results suggest that although the introduction of an alkyl group into the ligand is a good method to induce crystal deformation, the use of the alkyl chain does not always result in the induction of the crystal deformation.

We have reported a crystalline Co(II) complex that exhibits thermally induced, abrupt and reversible macroscopic shrinkage and expansion. In this complex, the *n*-butyls rotate reversibly in response to temperature as expected. Structural change at the molecular level expands to a macroscopic, abrupt crystal deformation through cooperative interaction in the crystal. The change in crystal size is 6–7% along the *c* axis, which is one of the largest reversible changes observed among metal–organic molecular crystals. Crystal structure analysis indicated that the π–π stacking pattern of rigid aromatic units enables the rotation of the *n*-butyl groups. The presence of strong and weak interactions in nearly orthogonal directions is responsible for the excellent resistance to fatigue. Thus, an alkyl chain can be used as a potential rotor for constructing thermally responsive crystalline materials, allowing for the potential use of this crystalline Co(II) complex as an actuator. This work provides a suitable model to understand the structure–property relationship between crystal deformation and alkyl rotation.

## Methods

### Materials

All reagents were obtained from commercial suppliers and were used without further purification. The ligands, *n*-butyl-2,6-di(1*H*-pyrazol-1-yl)isonicotinate (L), *n*-butyl-*d*_9_ 2,6-di(1*H*-pyrazol-1-yl)isonicotinate (L-*d*_9_), *n*-pentyl 2,6-di(1*H*-pyrazol-1-yl)isonicotinate (L1), *n*-hexyl 2,6-di(1*H*-pyrazol-1-yl)isonicotinate (L2) and *n*-decyl 2,6-di(1*H*-pyrazol-1-yl)isonicotinate (L3) were synthesized according to the literature methods, with minor modifications^32^.

### Synthesis of complex 1

The target complex [Co(NO_3_)_2_(L)] was prepared by layering an acetone solution of L (0.03 M, 5 cm^3^) on an acetone solution of Co(NO_3_)_2_·4H_2_O (0.03 M, 5 cm^3^) in a tube. The tube was sealed and left undisturbed at room temperature. X-ray-quality purple prismatic crystals appeared 2 days later in 66% yield. Analysed (calculated) (%) for C_16_H_17_CoN_7_O_8_: C, 38.98 (38.88); H, 3.46 (3.47); and N, 19.73 (19.84).

### Synthesis of complexes 2 and 2′

The analogue [Zn(NO_3_)_2_(L)] (**2**) was prepared by mixing an acetone solution of L (0.02 M, 5 cm^3^) with an acetone solution of Zn(NO_3_)_2_·6H_2_O (0.02M, 5 cm^3^) in a beaker under stirring for 2 min. The solution was filtered and allowed to slowly evaporate at room temperature to isolate crystalline solids with 52% yield. Analysed (calculated) (%) for C_16_H_17_ZnN_7_O_8_: C, 38.38 (37.98); H, 3.42 (3.43); and N, 19.58 (19.33). [Zn(NO_3_)_2_(L-*d*_9_)] (**2′**) was obtained in 55% yield via the same method used to prepare complex **2**. Analysed (calculated) (%) for C_16_H_8_D_9_ZnN_7_O_8_: C, 37.54 (37.70); and N, 19.13 (19.24).

### Synthesis of complexes 3–5

Complexes **3**–**5** were synthesized following the same synthetic procedure as that for complex **2** by mixing an acetone solution of L1/L2/L3 (0.02 M, 5 cm^3^) with an acetone solution of Co(NO_3_)_2_·4H_2_O (0.02 M, 5 cm^3^). Purple prismatic crystals were obtained (yield 68% for **3**, 55% for **4** and57% for **5**). Analysed (calculated) (%) for **3** (C_17_H_19_CoN_7_O_8_): C, 40.17 (39.97); H, 3.77 (3.72); and N, 19.29 (19.25); for **4** (C_18_H_23_CoN_7_O_9_): C, 40.01 (40.02); H, 4.29 (4.34); and N, 18.15 (18.11); for **5** (C_22_H_29_CoN_7_O_8_): C, 45.68 (44.34); H, 5.05 (5.10); and N, 16.95 (16.25).

### Single-crystal X-ray diffraction

Single-crystal X-ray data were collected on a Rigaku charge-coupled device diffractometer. A crystal was glued onto a nylon loop and enveloped in a temperature-controlled stream of dry nitrogen gas during data collection. The variable-temperature single-crystal data of complex **1** were repeatedly collected for the same crystal during cooling and heating. During cooling, single-crystal data were recorded at 303, 273, 238, 228, 183 and 123 K, however, during heating, single-crystal data were recorded at 183, 243, 253, 273 and 303 K (Supplementary Data 1). The single-crystal data of complexes **2**–**5** and **2′** were also collected (Supplementary Data 2 and 3). The structures were solved and refined by full-matrix least squares on *F*^2^ using the SHELX programme^33^ with anisotropic thermal parameters for all nonhydrogen atoms. Hydrogen atoms were added geometrically and refined using the riding model.

### Measurements

DSC measurements of the polycrystalline sample were performed on a Seiko EXSTAR 6000 instrument using cooling and heating rates of 10 K min^−1^. Heat capacity measurements of the polycrystalline sample were carried out in the temperature range between 7 and 300 K with a laboratory-made low-temperature adiabatic calorimeter^29^. The sample of 0.14947, g after buoyancy correction was loaded into a gold-plated copper cell and sealed together with helium gas at ambient pressure using an indium gasket. The helium gas functions as a heat exchange medium. Thermometry was performed with a rhodium–iron alloy resistance thermometer (nominal 27 Ω, Oxford Instruments) calibrated on the basis of the international temperature scale of 1990 (ITS-90). Infrared spectra were recorded using fine powder adhered to a CaF_2_ plate on a JASCO FT/IR-600 Plus spectrometer in the 400–4,000 cm^−1^ region. ^13^C-NMR spectra were measured on a JNM-LA400 NMR spectrometer (JEOL, Japan). Solid-state ^13^C-NMR spectra were measured with a CP/MAS probe. The sample (ca. 60 mg) was contained in a ceramic cylindrical rotor that was spun at 15 kHz. Solid-state ^2^H-NMR spectra were measured by a quadrupole echo pulse sequence *π*/2*x*–*τ*–*π*/2*y* on a Bruker DSX 300 spectrometer; the values for the *π*/2 pulse width and *τ* were and 2.0 and 20 μs and the repetition time was varied between 2 and 5 s. Direct current (DC) magnetic susceptibility measurements were performed on a MPMS-5S SQUID magnetometer in an applied field of 5,000 G over the whole temperature range.

### Computational methods

All calculations were performed with the DMol3 programme^34^,^35^ in Material Studio (Accelrys, Inc.). The Perdew–Burke–Ernzerhof generalized gradient functional was employed for the exchange-correlation energy. The wave functions were expanded in terms of numerical basis sets. We employed the DND basis set (double numerical basis set with the *d*-type polarization functions) for geometry optimization. The Brillouin zone was sampled with a (3 × 1 × 3) Monkhorst–Pack^36^ mesh of *k*-points. To reasonably describe weak interactions between the Co complexes, we used the dispersion correction method developed by Tkatchenko and Scheffler^37^. In addition to the atomic coordinates, the unit-cell parameters were optimized without symmetry constraints unless otherwise noted.

## Additional information

**Accession codes:** The X-ray crystallographic coordinates for structures reported in this article have been deposited at the Cambridge Crystallographic Data Centre (CCDC), under deposition numbers CCDC 1024026-1024036, CCDC 1407262-1407266, CCDC 1415773 and CCDC 1415774. These data can be obtained free of charge from The Cambridge Crystallographic Data Centre via www.ccdc.cam.ac.uk/data_request/cif.

**How to cite this article:** Su, S.-Q. *et al.* Assembling an alkyl rotor to access abrupt and reversible crystalline deformation of a cobalt(II) complex. *Nat. Commun.* 6:8810 doi: 10.1038/ncomms9810 (2015).

## Supplementary Material

Supplementary Information Supplementary Figures 1-18, Supplementary Tables 1-4 and Supplementary References

Supplementary Data 1 Variable-temperature single-crystal data of 1 recorded for the same crystal during cooling (303, 273, 238, 228, 183, and 123 K) and heating (183, 243, 253, 273, and 303 K)

Supplementary Data 2 Variable-temperature single-crystal data of **2** (153 and 303 K) and 2' (193 and 303 K)

Supplementary Data 3 Single crystal structures of **3-5** recorded at 123 K

Supplementary Movie 1 A crystal shrinking as the temperature was decreased at a rate of 5 K min^-1^ in the range 253-228 K. The crystal was glued to a nylon loop and enveloped in a temperature-controlled stream of dry nitrogen gas while the movie was recorded. This movie was recorded at normal speed.

Supplementary Movie 2 A crystal expanding as the temperature was increased at a rate of 5 K min^-1^ in the range 228-253 K. The crystal was glued to a nylon loop and enveloped in a temperature-controlled stream of dry nitrogen gas while the movie was recorded. This movie was recorded at normal speed.
