# Supplementary material for: Assembling an alkyl rotor to access abrupt and reversible crystalline deformation of a cobalt(II) complex
Source: Nat Commun. 2015 Nov 4;6:8810. doi: 10.1038/ncomms9810 (PMC4667628; doi:10.1038/ncomms9810)
Supplement: Supplementary Information — Supplementary Figures 1-18, Supplementary Tables 1-4 and Supplementary References [file ncomms9810-s1.pdf]

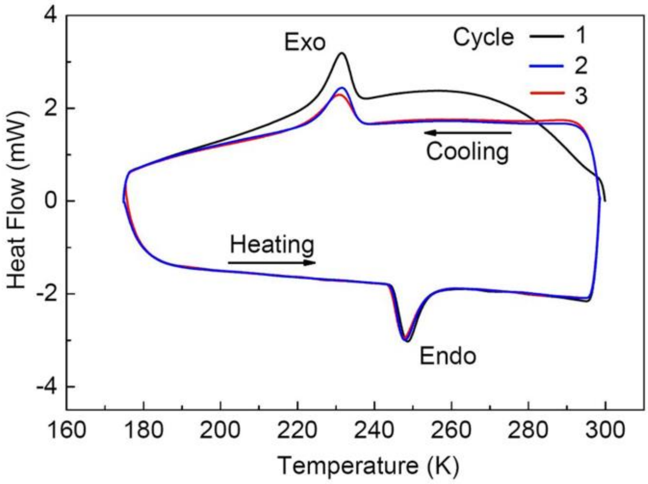

Supplementary Figure 1 | Effect of thermal cycling on the phase transition in crystal **1**. DSC curves recorded at the rate of 10 K min^{-1}. Over three cycles, the sample behaved in an identical way and almost no changes in the phase-transition temperatures were observed.

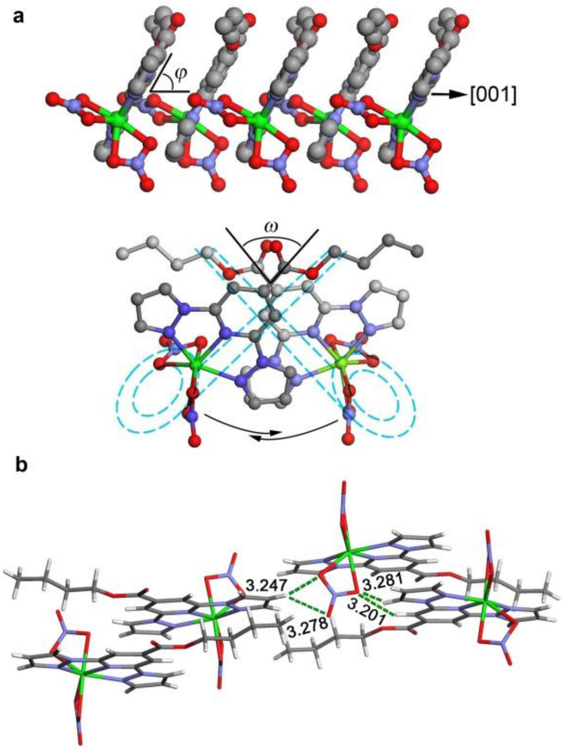

Supplementary Figure 2 | Molecular stacking and interactions in the crystal **1** (low-temperature phase). (**a**) Atoms are shown in ball-and-stick representation. A perspective view of the columnar packing in the scissor-crossover mode. The staggered molecules stack in parallel with the molecular planes tiled at an angle φ of 61.65° relative to the [001] direction and the cross angle ω of 74.93°. The blue dotted portion is the outline of the scissors. (**b**) 3D molecular packing viewed along [−1 0 1] is mainly stabilized by C–H···O interactions at distances of 3.201–3.281 Å. Green, Co; gray, C; white, H; blue, N; and red, O.

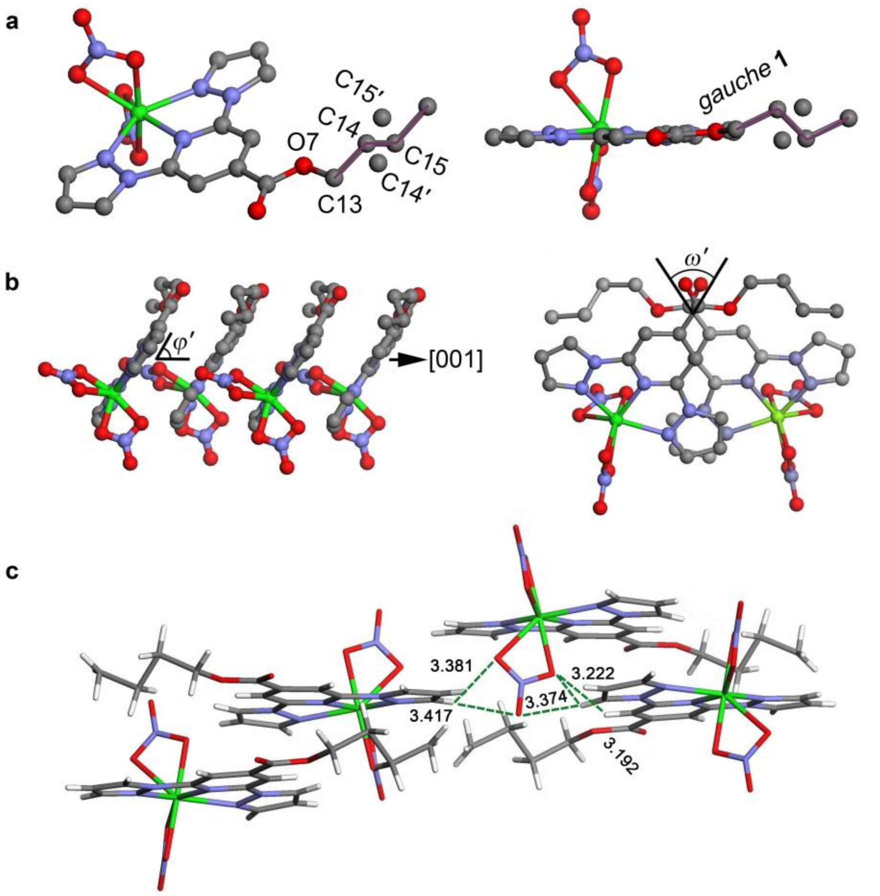

Supplementary Figure 3 | Molecular structure, stacking and interactions in the crystal of **1** (high-temperature phase). (**a**) The most reasonable *gauche* conformation, *gauche* **1** (purple), in the high-temperature phase. Two methylene carbon atoms of the *n*-butyl are disordered (C14, C14′, C15, and C15′). We should note that the value of torsion angle O(7)-C(13)-C(14)-C(15) in *gauche* **1** is not strictly 60° (Supplementary Table S2). (**b**) A perspective view of columnar packing in the scissor-crossover mode. The staggered molecules stack in parallel with the molecular planes tilted at an angle φ' of 60.25° relative to the [001] direction and at a cross angle ω' of 65.73°. Hydrogen and disordered carbon atoms (C14′ and C15′) are omitted for clarity. **c**, 3D molecular packing viewed along [−1 0 1] is mainly stabilized by C–H···O interactions at distances from 3.192 to 3.417 Å. Green, Co; gray, C; white, H; blue, N; and red, O.

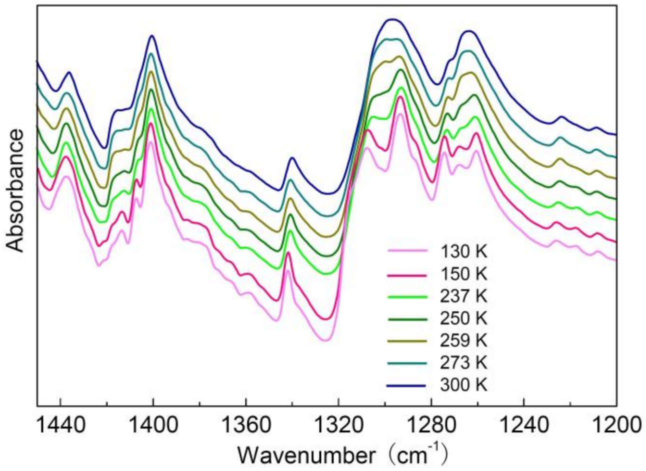

Supplementary Figure 4 | Variable-temperature infrared absorbance spectra of **1** in the 1450 to 1200 cm^{-1} region. In the spectrum of the low-temperature phase, the two peaks at 1307 and 1293 cm^{-1}, which correspond to the CH_2 wagging mode, are well identified. As the temperature is increased, the peak at 1307 cm^{-1} becomes unrecognizable at temperatures near the phase-transition temperature; in the spectrum of the high-temperature phase, a broad peak is observed instead of the original two peaks. The smearing effect of the peaks over the region (1322–1278 cm^{-1}) suggests that, at around the phase transition temperature, the *n*-butyl groups become highly disordered[1-3].

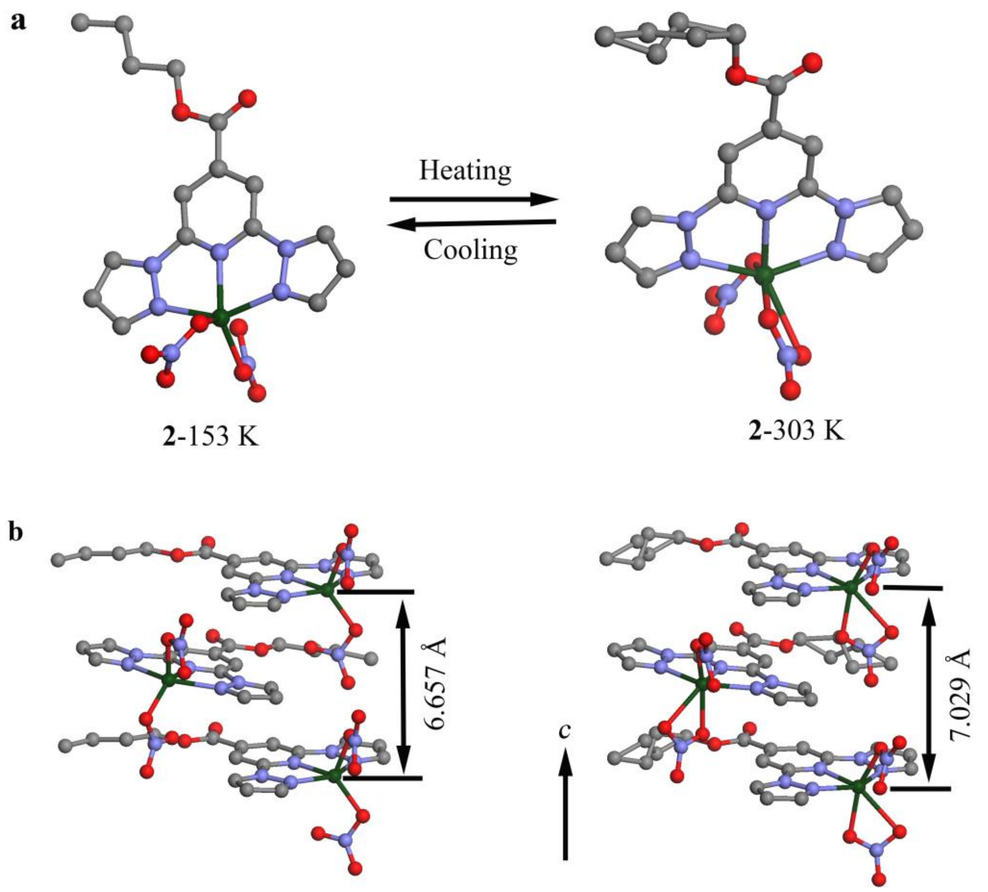

Supplementary Figure 5 | Molecular structures of crystal **2** at 153 and 303 K. X-ray data of **1** shows that the ligand L has disorder. However, the X-ray data are unable to confirm whether the disorder is static or dynamic. Therefore, in order to measure solid-state ^{13}C CP/MAS NMR, we synthesized $[Zn(NO_3)_2(L)]$ (complex **2**) as an analog to **1**, where the Co^{2+} is replaced with diamagnetic Zn^{2+}. The complex **2** exhibits phase transition at approximately 175 K (see **Supplementary Figure 6**), wherein structural change in **2** (**Supplementary Table 2**) is similar to that in **1**. (**a**) Two isomers could be reversibly transformed with rotation of the *n*-butyl group. At the high temperature, two methylene carbon atoms of the *n*-butyl are disordered. (**b**) The molecules stack in parallel along the *c*-axis with different distances between the molecules at the low- and high-temperature. Hydrogen atoms are omitted for clarity. Olive, Zn; gray, C; blue, N; and red, O. The solid-state ^{13}C CP/MAS NMR data is shown in **Supplementary Figure 7**.

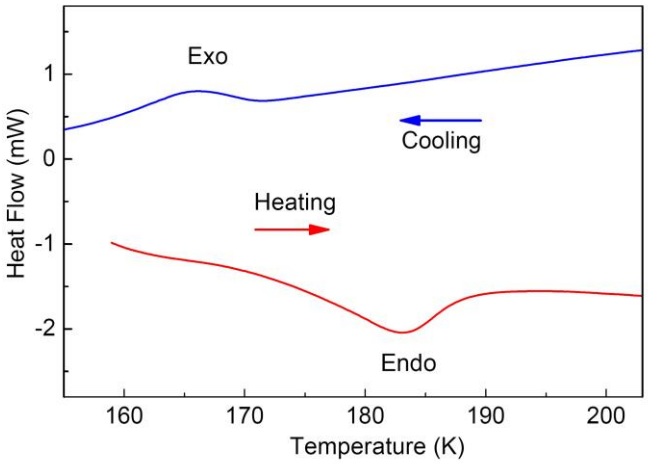

Supplementary Figure 6 | Temperature dependence of differential scanning calorimetry (DSC) for **2**. DSC curves of crystal **2** recorded at the rate of 10 K min^{-1} during a cooling–heating (blue–red lines) cycle. The DSC curves for the crystals of **2** exhibit a single exothermic peak at 166 K during the cooling process. In contrast, the DSC traces of **2** exhibit an endothermic peak at 183 K during the heating process. Exo: exothermic peak; Endo: endothermic peak.

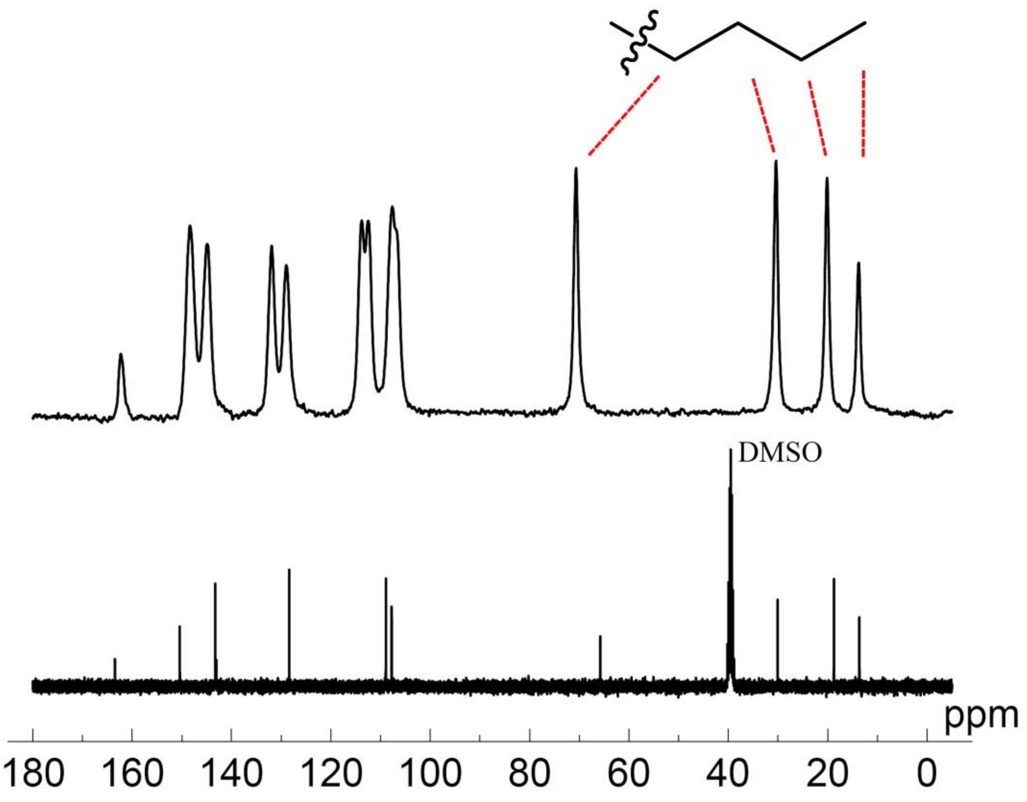

Supplementary Figure 7 | (Top) solid-state (CP/MAS) and (bottom) solution (DMSO-d_6) ^{13}C NMR spectra of **2** at room temperature. The spectrum is highly resolved, each nonequivalent carbon giving rise to a resonance, suggesting that the disorder in **2** is dynamic. This suggests that the disorder in **1** is dynamic. This conclusion is supported by temperature variation of the deuterium NMR spectrum of partially deuterated **2** (**Supplementary Figures 8–11**).

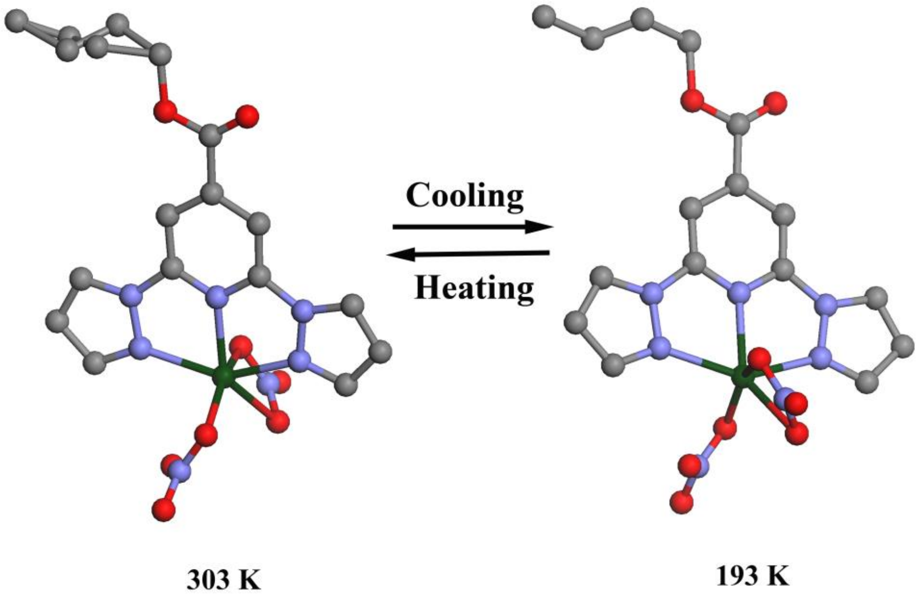

Supplementary Figure 8 | Molecular structures of **2′** at 303 and 193 K during the cooling process. Hydrogen and deuterium atoms are omitted for clarity. Olive, Zn; gray, C; blue, N; and red, O.

To measure the ^{2}H NMR spectrum, we synthesized deuterated [Zn(NO_3)$_2$(L-d_9)] (complex **2′**). Complex **2′** exhibits a phase transition at approximately 206 K (see **Supplementary Figure 9**). Unfortunately, the structural change in **2′** (**Supplementary Table 3**) differs from those in **1** and **2**. However, the structure of **2′** at high temperature phase (303 K) is similar to those of complexes **1** and **2** with a disordered butyl group. Therefore, we measured the solid-state ^{2}H NMR spectrum of **2′** to characterize the disorder of the *n*-butyl group (**Supplementary Figure 10**).

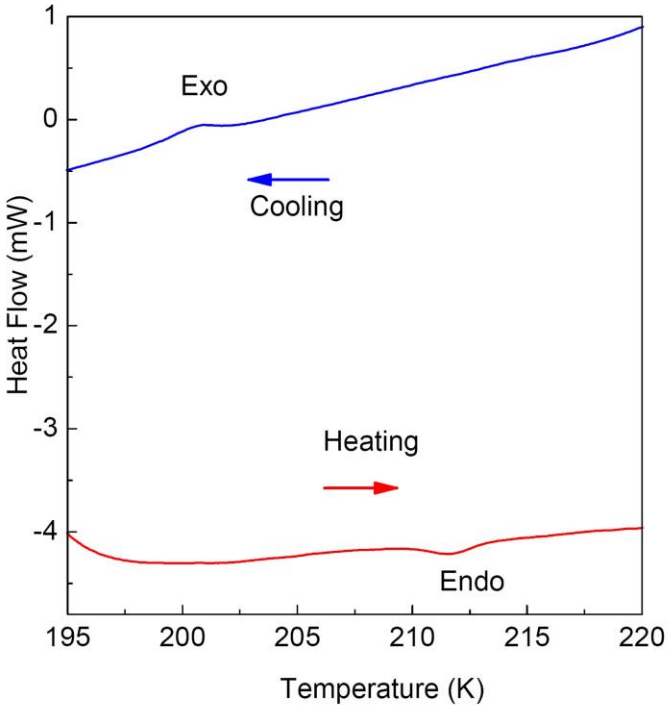

Supplementary Figure 9 | Temperature dependence of differential scanning calorimetry (DSC) for **2′**. DSC curves for crystal **2′** recorded at 5 K min^{-1} during a cooling–heating (blue–red lines) cycle. The DSC curves for the crystals of **2′** exhibit a single exothermic peak at 201 K during the cooling process (220–195 K). In contrast, the DSC traces of **2′** exhibit an endothermic peak at 212 K during the heating process (195–220 K). Exo: exothermic peak; Endo: endothermic peak.

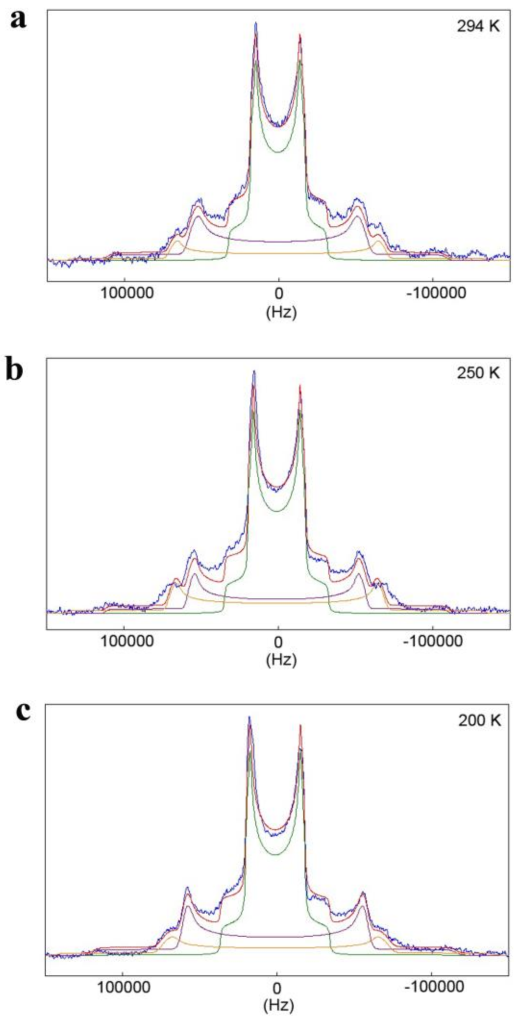

Supplementary Figure 10 | Temperature variation of the deuterium NMR spectrum of *n*-butyl-d_9 group, (**a**) 294 K, (**b**) 250 K, and (**c**) 200 K. Concrete evidence of the dynamic disorder in **2** was obtained from the solid-state ^{2}H NMR spectrum of partially deuterated **2** (complex **2′**). The observed spectra were analyzed by a DmFit program and apparent quadrupole parameters were obtained[4]. Three spectral components with different motional amplitudes of C–D bonds were distinguished between 294 and 200 K, shown as blue: observed, green: terminal methyl group, purple: two central methylene groups, orange: methylene-group-linked oxygen atom, and red: sum of the three components.

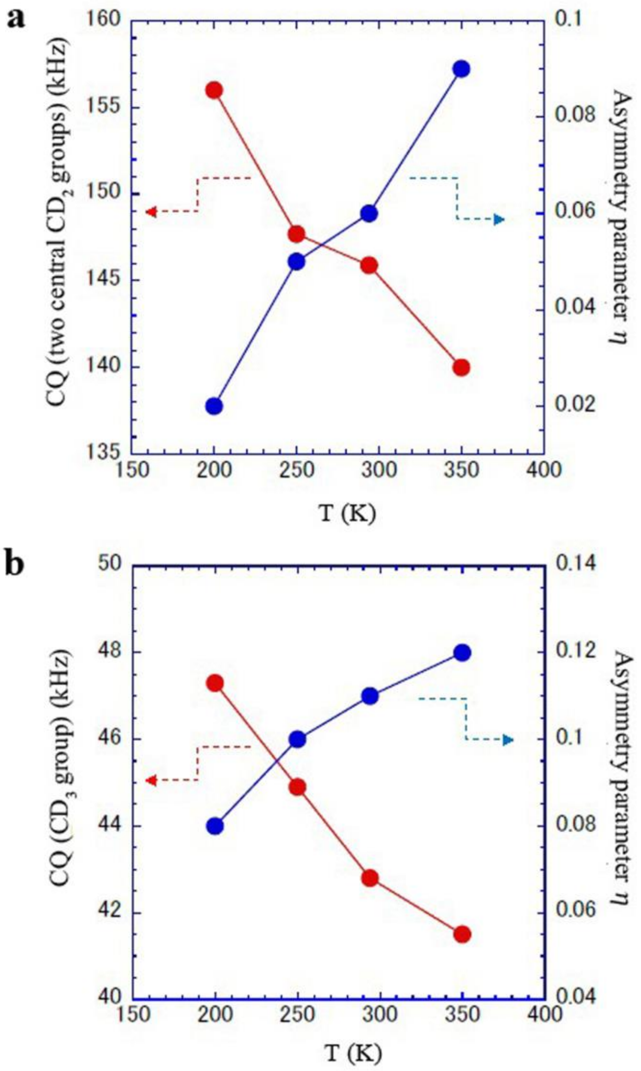

Supplementary Figure 11 | Temperature variation of the quadrupole parameters, quadrupole coupling constant CQ and asymmetry parameter η of (**a**) two central methylene groups and (**b**) the terminal methyl group. Both figures (a) and (b) show a decrease of the effective CQ and an increase of η as temperature was increased, indicating a change in the angle of conical motion of directional vectors of C–D bonds of two central methylene groups and in the angle of conical motion of the C_3 rotation axis of the rapidly rotating terminal methyl group under rapid-motion limit. Defining the angle θ between the axis of the cone and it's slant, two possible values of θ were estimated for both motions to be 15–20°or 165–160° between 200 and 350 K by simulation

of ^{2}H-NMR spectra[5,6], The motion of C_3 rotation axis of the terminal methyl group was dragged by the motion of the two flexible central methylene groups. Typical quadrupole coupling parameters of the deuterium atom forming a C–D covalent bond are known to be CQ = 170–180 kHz and η = 0.02 for the static condition, which are almost independent of methyl or methylene group. The effective quadrupole coupling parameters are sensitive to motion of the direction of C-D bond[7] and CQ value of the rapidly rotating methyl group is *ca*. 52 kHz. C–D bonds of carbon atoms directly linked to an oxygen atom are almost static and their CQ values are close to 180 kHz (**Supplementary Figure 10**).

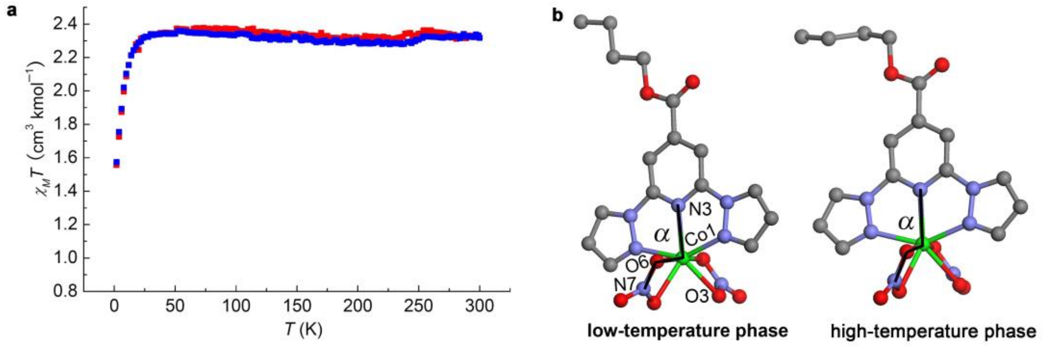

Supplementary Figure 12 | Magnetic anomaly and the changes in the coordination environment of Co(II) ion. (**a**) Temperature dependence of magnetic susceptibility for **1** under cooling (red) and heating modes (blue). A step in the magnetic susceptibility is observed near the phase-transition temperature. (**b**) After the phase transition, the dihedral angle α and the bond length of Co1–O3 change significantly because the nitrate ions are twisted (low-temperature phase: α, 143.43°; Co1–O3, 2.198 Å; high-temperature phase: α, 150.40°; Co1–O3, 2.342 Å). Gray, C; blue, N; and red, O.

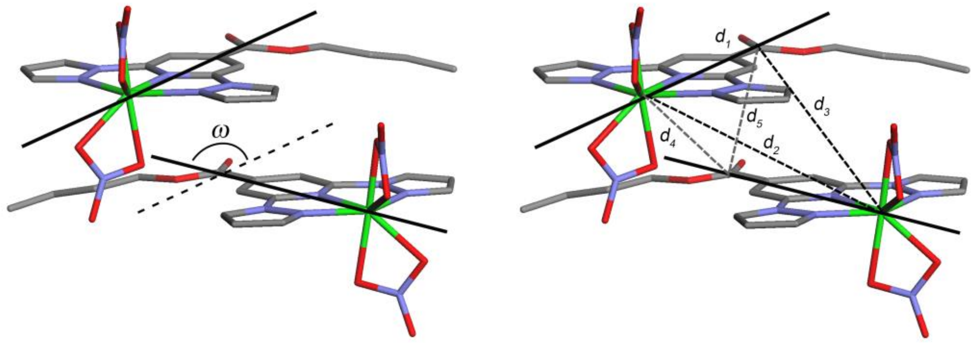

Supplementary Figure 13 | The crossing angle ω (ω = 74.93°) of the adjacent molecules arranged in the mode of scissor-crossover. The value of ω can be calculated by the cosine theorem (simplified formula, $\sin [(\pi - \omega)/2] = (2d_1^2 - d_2^2 + d_3^2 + d_4^2 - d_5^2)^{1/2} / 2d_1$). The distances between atoms (d_1–d_5) can be measured directly (d_1, 6.403 Å; d_2, 7.783 Å; d_3, 8.241 Å; d_4, 5.402 Å; d_5, 3.900 Å). Green, Co; gray, C; blue, N; and red, O.

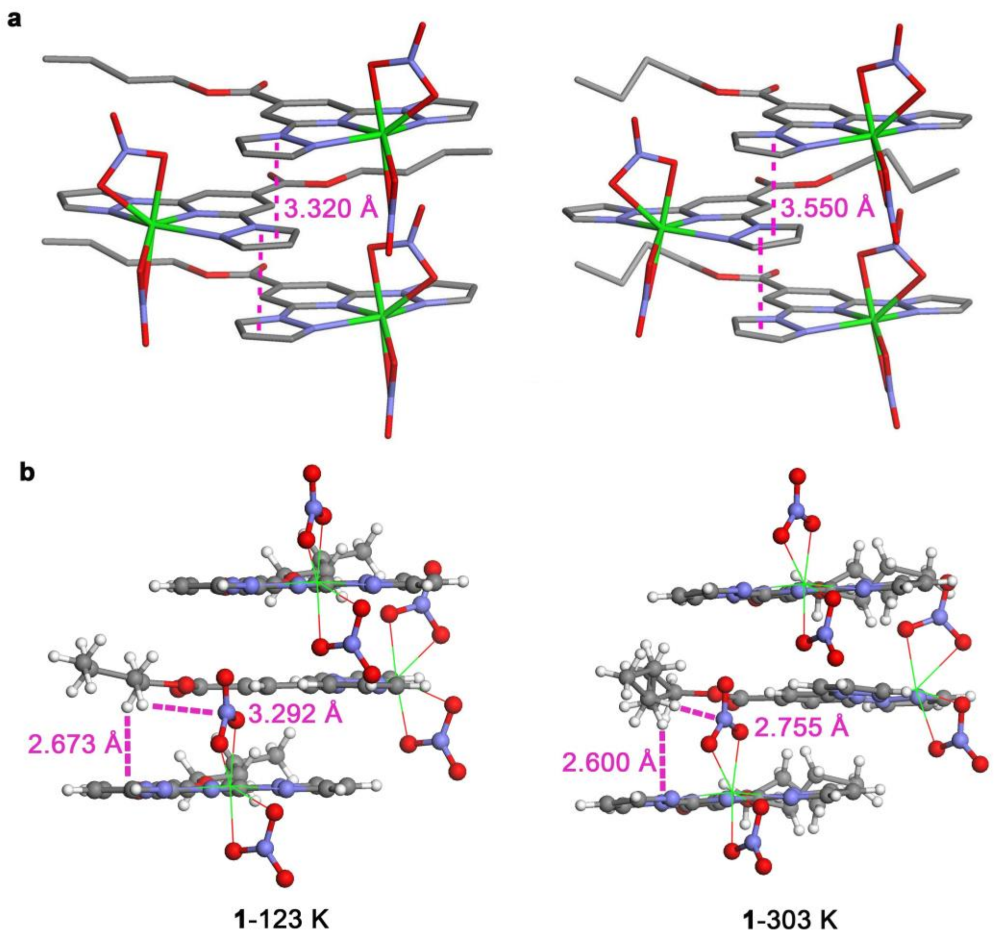

Supplementary Figure 14 | The interactions and distances between adjacent molecules along the *c*-axis in the two phases of the crystal of **1**. (**a**) The *π*–*π* interactions and distances between adjacent molecules. In complex **1**, the molecules are the large conjugated systems, and the molecules are arranged in a parallel-displaced geometry along the *c*-axis. (**b**) The shortest distances between the *n*-butyl group and the molecular planes of adjacent molecules are 2.673 and 2.600 Å in the low-temperature and high-temperature phase separately. Between the *n*-butyl group and the coordinated nitrate ions of adjacent molecules, the shortest distances are 3.292 and 2.755 Å (the distance of H···N) in the low-temperature and high-temperature phase separately. Green, Co; gray, C; blue, N; and red, O.

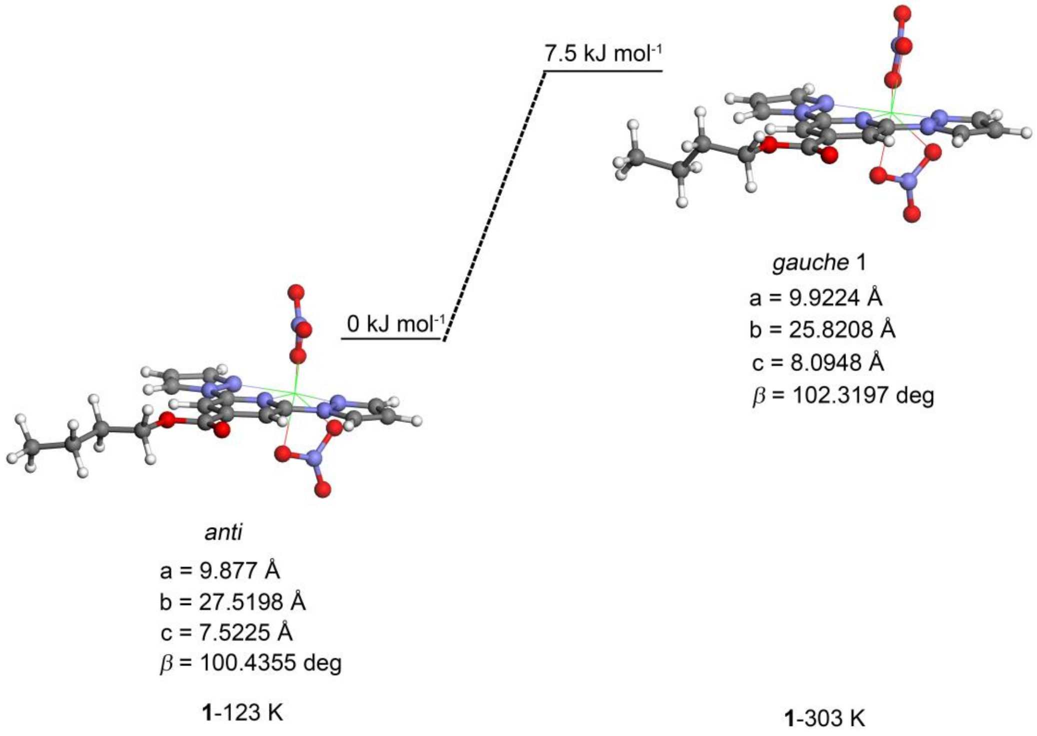

Supplementary Figure 15 | Optimized structures and energies for the *anti* and *gauche* conformations of **1** (high-temperature phase and low-temperature phase). The structure in the *anti* conformation is 7.5 kJ mol^{-1} more stable than that in the *gauche* **1**conformation. Green, Co; gray, C; white, H; blue, N; and red, O.

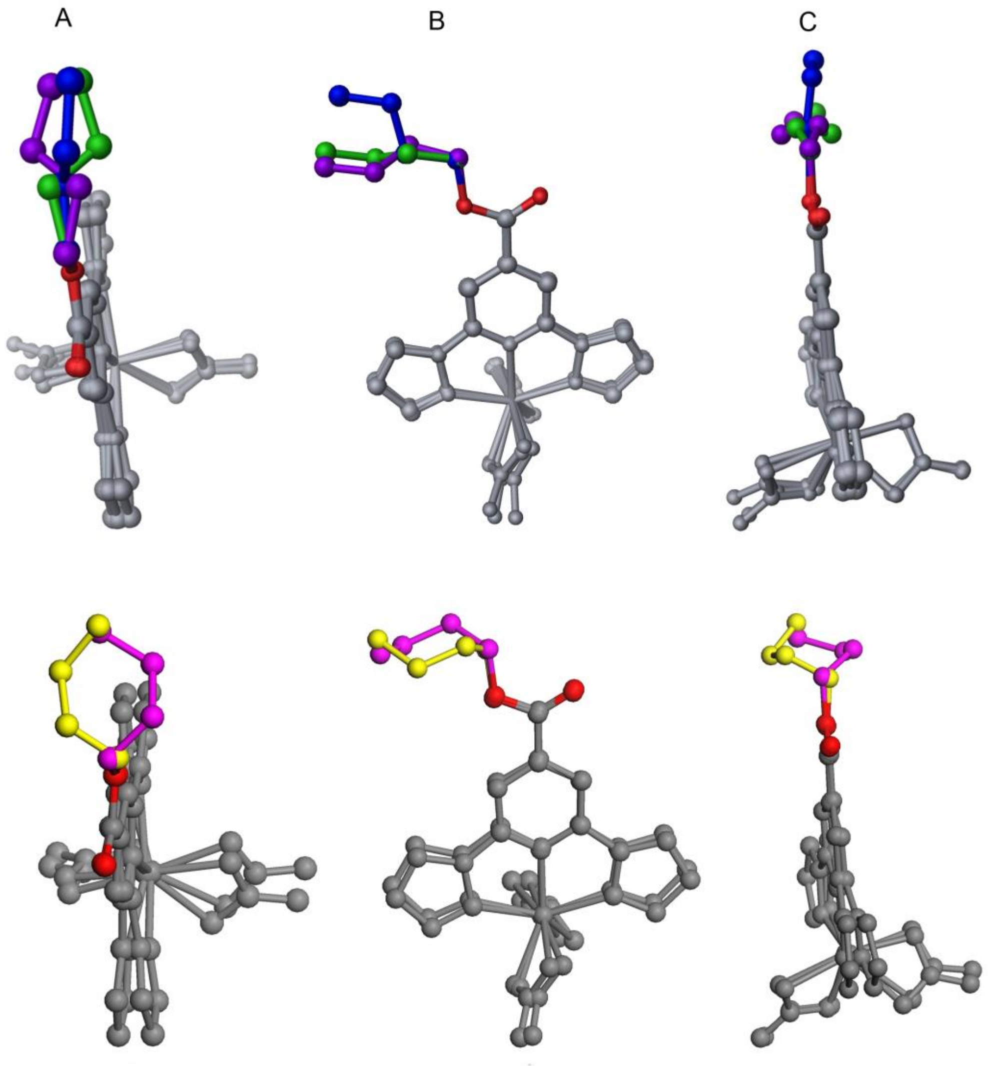

Supplementary Figure 16 | Top (A) and side (B and C) views of superimposition of optimized structures of **1** in the *anti* and *gauche* conformations. Because two methylene carbon atoms (C14 and C15) are disordered in the high-temperature phase and each atom can occupy either of the two possible sites, three conformations, which are noted here as *gauche* **1a**, **1b** and **1c**, other than *gauche* **1** can be considered on the basis of the single-crystal analysis of the high-temperature phase. These optimized structures *gauche* **1a**, **1b** and **1c** were obtained under the assumption that each structure is in a 100 % in the crystal. Blue, *anti*; green, *gauche* **1**; purple, *gauche* **1a**; pink, *gauche* **1b**; yellow, *gauche* **1c**; and red, oxygen atoms.

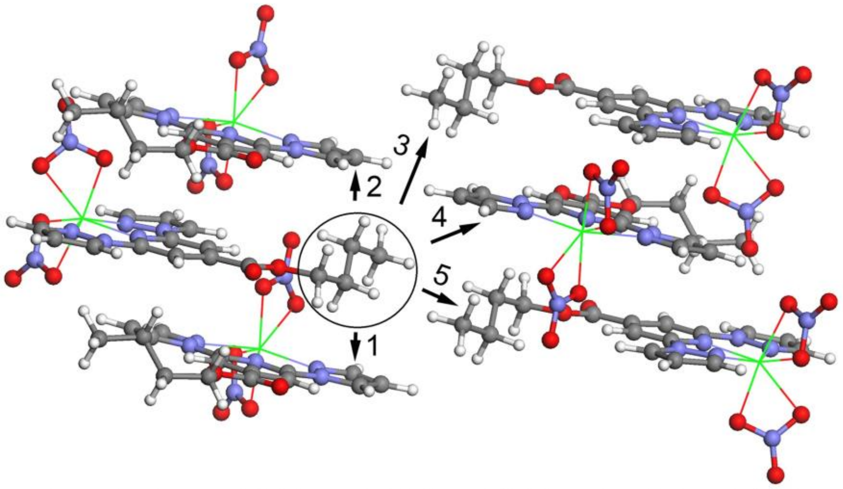

ΔE = − 9.4 kJ mol^{-1} (1); − 1.8 kJ mol^{-1} (2); − 0.4 kJ mol^{-1} (3); 3.8 kJ mol^{-1} (4); 3.3 kJ mol^{-1} (5)

Supplementary Figure 17 | Repulsive interactions calculated between the *n*-butyl group in the *gauche* conformation and the surrounding Co^{II} complexes. ΔE = E(expand in c-direction) – E(contract in c-direction), where E(expand in c-direction) is the repulsive interaction in the expanded unit cell obtained by relaxing the unit-cell parameters and E(contract in c-direction) is the repulsive interaction in the contracted structure obtained by keeping the unit-cell parameters fixed at the *anti* conformation. The negative values indicate that the repulsive interactions are reduced by the expansion of the unit cell in the c-direction. These values were obtained using the OPLS 2005 force field. Green, Co; gray, C; white, H; blue, N; and red, O.

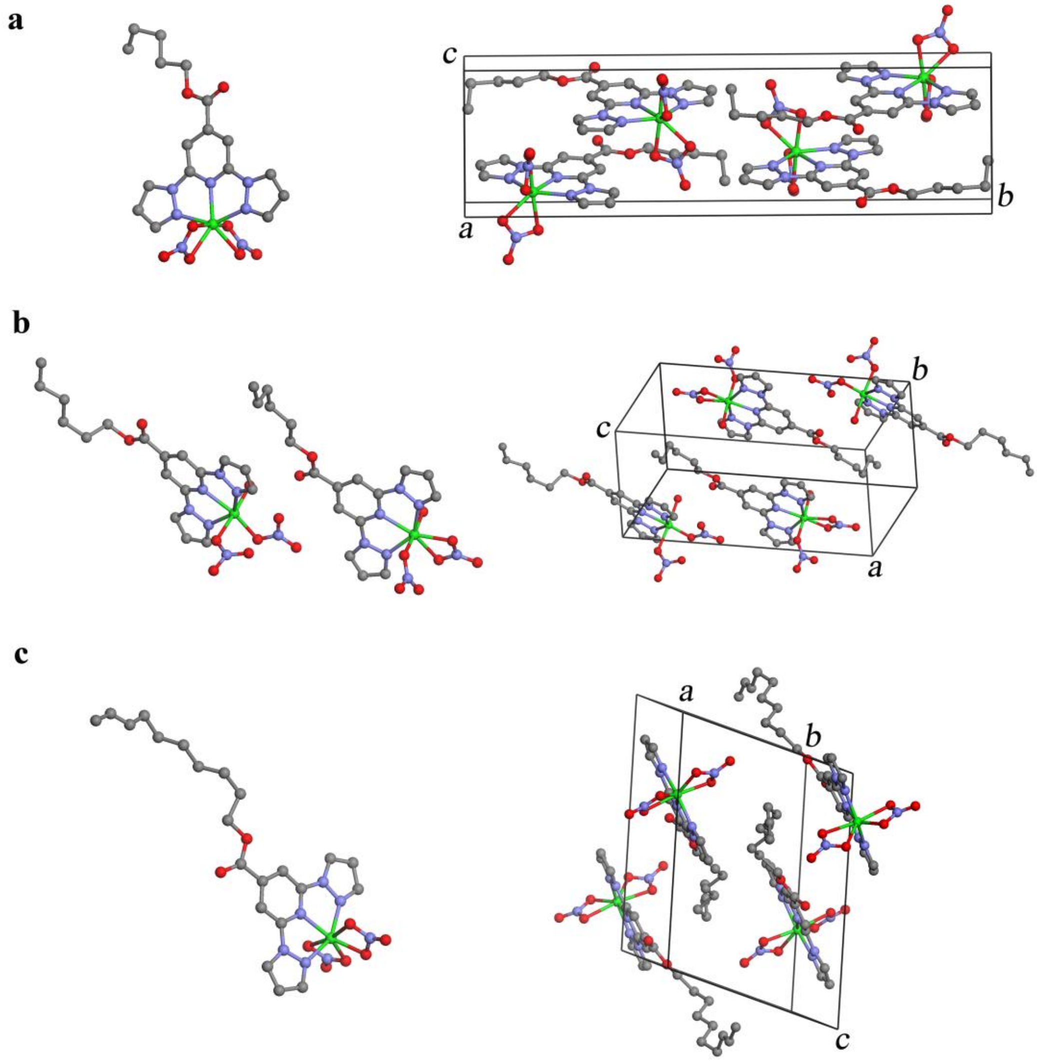

Supplementary Figure 18 | Molecular structures and stacking in the crystals of (**a**) **3**, (**b**) **4**, and (**c**) **5** at 123 K. The molecules in crystal **3** stack in parallel with the scissor-crossover mode along the *c*-axis, as in crystal **1**. The molecular stacking in crystals **4** and **5** is completely different from in crystal **1**. Compared to the *anti* conformation of the butyl group in crystal **1**, the conformations of alkyl chains in crystals **3-5** are different and multiple. Atoms are shown in ball-and-stick representation. Green, Co; gray, C; white, H; blue, N; and red, O.

Supplementary Table 1 | Table of crystallographic parameters for complex **1**.

	Complex **1** (on cooling)					
T (K)	303	273	238	228	183	123
Formula	$CoC_{16}H_{17}N_7O_8$	$CoC_{16}H_{17}N_7O_8$	$CoC_{16}H_{17}N_7O_8$	$CoC_{16}H_{17}N_7O_8$	$CoC_{16}H_{17}N_7O_8$	$CoC_{16}H_{17}N_7O_8$
Formula weight	494.30	494.30	494.30	494.30	494.30	494.30
Crystal system	Monoclinic	Monoclinic	Monoclinic	Monoclinic	Monoclinic	Monoclinic
Space group	$P2_1/c$	$P2_1/c$	$P2_1/c$	$P2_1/c$	$P2_1/c$	$P2_1/c$
a (Å)	9.8533(13)	9.8490(11)	9.8458(15)	9.7984(18)	9.7948(18)	9.794(3)
b (Å)	25.995(3)	25.942(3)	25.898(4)	27.410(5)	27.435(5)	27.475(9)
c (Å)	8.1782(10)	8.1610(9)	8.1434(14)	7.6404(16)	7.5979(15)	7.548(3)
β (deg)	100.741(2)	100.764(2)	100.839(3)	100.082(4)	100.265(5)	100.433(8)
V ($Å^3$)	2058.1(5)	2048.5(4)	2039.4(6)	2020.3(7)	2009.0(7)	1997.5(11)
Z	4	4	4	4	4	4
D_{calcd} (g cm^{-3})	1.595	1.603	1.610	1.625	1.634	1.644
F(000)	1012	1012	1012	1012	1012	1012
data collected	17031	16941	16901	16695	15678	16278
unique data	3810	3763	3742	3723	3656	3672
R(int)	0.0492	0.0284	0.0283	0.0312	0.0487	0.0375
GOF on F^2	1.310	1.081	1.062	1.086	1.192	1.053
R_1[a] [I > 2σ(I)]	0.0728	0.0335	0.0310	0.0309	0.0470	0.0301
ωR_2[b](all data)	0.1663	0.0845	0.0808	0.0776	0.2094	0.0728

[a] $R_1 = \Sigma||F_o|-|F_c||/\Sigma|F_o|$. [b] $\omega R_2 = \{\Sigma[\omega(F_o^2-F_c^2)^2]/\Sigma[\omega(F_o^2)^2]\}^{1/2}$

	Complex **1** (on heating)				
T (K)	183	243	253	273	303
Formula	$CoC_{16}H_{17}N_7O_8$	$CoC_{16}H_{17}N_7O_8$	$CoC_{16}H_{17}N_7O_8$	$CoC_{16}H_{17}N_7O_8$	$CoC_{16}H_{17}N_7O_8$
Formula weight	494.30	494.30	494.30	494.30	494.30
Crystal system	Monoclinic	Monoclinic	Monoclinic	Monoclinic	Monoclinic
Space group	$P2_1/c$	$P2_1/c$	$P2_1/c$	$P2_1/c$	$P2_1/c$
a (Å)	9.796(2)	9.804(4)	9.8515(18)	9.865(2)	9.8591(12)
b (Å)	27.433(6)	27.417(10)	25.892(5)	25.934(6)	26.027(3)
c (Å)	7.5962(17)	7.659(3)	8.1511(15)	8.172(2)	8.1886(10)
β (deg)	100.288(5)	100.030(5)	100.820(2)	100.762(3)	100.676(3)
V (Å^3)	2008.5(7)	2027.1(13)	2042.2(6)	2053.8(9)	2064.8(4)
Z	4	4	4	4	4
D_{calcd} (g cm^{-3})	1.635	1.620	1.608	1.599	1.590
F(000)	1012	1012	1012	1012	1012
data collected	15991	14242	15133	15006	17707
unique data	3666	4582	4548	4578	3818
R(int)	0.0488	0.0393	0.0321	0.0346	0.0627
GOF on F^2	1.127	0.972	1.020	0.970	1.180
$R_1{}^a$ [I > 2σ(I)]	0.0328	0.0321	0.0308	0.0323	0.0691
$\omega R_2{}^b$(all data)	0.0869	0.0873	0.0845	0.0888	0.1317

[a] $R_1 = \Sigma||F_o|-|F_c||/\Sigma|F_o|$. [b] $\omega R_2 = \{\Sigma[\omega(F_o{}^2-F_c{}^2)^2]/\Sigma[\omega(F_o{}^2)^2]\}^{1/2}$

Supplementary Table 2 | Table of crystallographic parameters for complex **2–5**.

	Complex **2**		Complex **3**	Complex **4**	Complex **5**
T (K)	153	303	123	123	123
Formula	$C_{16}H_{17}N_7O_8Zn$	$C_{16}H_{17}N_7O_8Zn$	$C_{17}H_{19}CoN_7O_8$	$C_{18}H_{23}CoN_7O_9$	$C_{22}H_{29}CoN_7O_8$
Formula weight	500.74	500.74	508.32	540.36	578.45
Crystal system	Monoclinic	Monoclinic	Monoclinic	Triclinic	Monoclinic
Space group	$P2_1/c$	$P2_1/c$	$P2_1/c$	*P*-1	P21/c
a (Å)	9.801(2)	9.915(2)	9.699(3)	8.0370(16)	11.000(2)
b (Å)	27.120(5)	26.133(5)	27.857(9)	15.043(3)	16.030(3)
c (Å)	7.7592(16)	8.3197(17)	7.707(2)	18.574(4)	15.347(3)
α (deg)	90	90	90	90.537(6)	90
β (deg)	101.09(3)	101.90(3)	101.440(9)	94.769(5)	106.744(5)
γ (deg)	90	90	90	92.761(4)	90
V (Å^3)	2023.8(7)	2109.4(7)	2040.9(11)	2235.1(7)	2591.4(9)
Z	4	4	4	4	4
D_{calcd} (g cm^{-3})	1.643	1.577	1.654	1.606	1.483
F(000)	1024	1024	1044	1116	1204
data collected	16508	19121	18020	19150	23496
unique data	4475	4682	4595	8207	5361
R(int)	0.0549	0.0524	0.0573	0.0279	0.0418
GOF on F^2	1.011	1.038	1.043	1.095	1.116
R_1[a] [I > 2σ(I)]	0.0533	0.0579	0.0426	0.0406	0.0833
ωR_2[b](all data)	0.1332	0.1578	0.0898	0.0983	0.2006

[a] $R_1 = \Sigma||F_o|-|F_c||/\Sigma|F_o|$. [b] $\omega R_2 = \{\Sigma[\omega(F_o^2-F_c^2)^2]/\Sigma[\omega(F_o^2)^2]\}^{1/2}$

Supplementary Table 3 | Table of crystallographic parameters for complex **2′**.

	Complex **2′** (on cooling)	
T (K)	303	193
Formula	$C_{16}H_8D_9N_7O_8Zn$	$C_{16}H_8D_9N_7O_8Zn$
Formula weight	509.74	509.74
Crystal system	Monoclinic	Monoclinic
Space group	$P2_1/c$	$P2_1/c$
a (Å)	9.871(2)	9.860(4)
b (Å)	26.022(6)	25.836(10)
c (Å)	8.286(2)	8.226(3)
β (deg)	101.902(6)	102.973(10)
V (Å^3)	2082.7(8)	2041.9(13)
Z	4	4
D_{calcd} (g cm^{-3})	1.597	1.629
F(000)	1024	1024
data collected	19070	18211
unique data	4679	4580
R(int)	0.0683	0.0749
GOF on F^2	1.062	1.022
R_1[a] [I > 2σ(I)]	0.0599	0.0590
ωR_2[b](all data)	0.1700	0.1424

[a] $R_1 = \Sigma||F_o|-|F_c||/\Sigma|F_o|$. [b] $\omega R_2 = \{\Sigma[\omega(F_o^2-F_c^2)^2]/\Sigma[\omega(F_o^2)^2]\}^{1/2}$

Supplementary Table 4 | Table of selected bond distances (Å) and torsion angles (°) in the low- and high-temperature phases of complex **1**.

**1** (123 K)		**1** (303 K)	
Co(1)-N(3)	2.1462(15)	Co(1)-N(3)	2.139(2)
Co(1)-O(1)	2.1585(14)	Co(1)-O(1)	2.117(2)
Co(1)-O(6)	2.1563(14)	Co(1)-N(1)	2.144(2)
Co(1)-N(1)	2.1651(16)	Co(1)-O(6)	2.174(2)
Co(1)-N(5)	2.1824(16)	Co(1)-N(5)	2.189(2)
Co(1)-O(4)	2.2188(16)	Co(1)-O(4)	2.163(3)
Co(1)-O(3)	2.1977(14)	Co(1)-O(3)	2.342(3)
N(3)-Co(1)-O(6)-N(7)	143.43(10)	N(3)-Co(1)-O(6)-N(7)	150.40(18)
N(3)-Co(1)-O(1)-N(6)	149.03(10)	N(3)-Co(1)-O(1)-N(6)	147.72(19)
O(7)-C(13)-C(14)-C(15)	171.44(15)	O(7)-C(13)-C(14)-C(15)	71.6(7)

Supplementary References

1. Sorai, M., Tsuji, K., Suga, H. & Seki, S. Studies on disc-like molecules I. Heat capacity of benzene hexa-n- hexanoate from 13 to 393 K. Mol. *Cryst. Liq. Cryst*. **59**, 33–58 (1980).

2. Maroncelli, M., Qi, S. P., Strauss, H. L. & Snyder, R. G. Nonplanar conformers and the phase behavior of solid n- alkanes. *J. Am. Chem. Soc*. **104**, 6237–6247 (1982).

3. Sorai, M. & Saito, K. Alkyl chain acting as entropy reservoir in liquid crystalline materials. *Chem. Rec*. **3**, 29–39 (2003).

4. Massiot, D. *et al*. Modelling one- and two-dimensional solid state NMR spectra. *Magn. Reson. Chem.* **40**, 70–76 (2002).

5. Takeda, S. *et al*. Restricted rotational motion of interlayer water molecules in vanadium pentoxide hydrate, $V_2O_5 \cdot nD_2O$, as studied by deuterium NMR. *Z. Naturforsch*. **57 a**, 419–424 (2002).

6. Sidhu, P. S. *et al*. A deuterium NMR study of guest molecular dynamics in tris(5-acetyl-3-thienyl) methane inclusion compounds. *Can. J. Chem.* **74**, 1784-1794 (1996).

7. Tycko, R. *Nuclear Magnetic Resonance Probes of Molecular Dynamics*. Kluwer Academic Publishers, the Netherlands, ISMN 0-7923-2795-0 (1994).
